# High Storable Power Density of Triboelectric Nanogenerator within Centimeter Size

**DOI:** 10.3390/ma16134669

**Published:** 2023-06-28

**Authors:** Yurui Shang, Chengyu Li, Gao Yu, Yuhan Yang, Wenting Zhao, Wei Tang

**Affiliations:** 1 School of Electrical Engineering, Guangxi University, Nanning 530004, China; shangyurui@binn.cas.cn; 2 Beijing Institute of Nanoenergy and Nanosystems, Chinese Academy of Sciences, Beijing 100083, China; lichengyu@binn.cas.cn (C.L.); yugao@binn.cas.cn (G.Y.); yangyuhan@binn.cas.cn (Y.Y.); 3 School of Mechanical and Electrical Engineering, University of Electronic Science and Technology of China, Chengdu 611731, China; 202121040206@std.uestc.edu.cn; 4 Energy Storage and Electrotechnics Department, China Electric Power Research Institute, Beijing 100192, China

**Keywords:** triboelectric nanogenerator, storable power density, power management circuit

## Abstract

Triboelectric nanogenerators (TENGs) possess significant attributes, such as a simple structure, high energy conversion efficiency, and ease of fabrication, rendering them crucial for powering mobile and distributed low-power electronic devices. In this study, a multilayer spring TENG with a cushion layer structure is proposed that enhances the output performance of the basic TENG structure. The fundamental topology of the energy harvesting circuit is chosen based on the electrical performance parameters of the generator and optimizes the selection of each electronic component in the actual circuit. This allows the small-size TENG (2 cm^3^) to have a high storable power density (5.45 mW m^−2^). Finally, the fabrication method of the small-size TENG and how to choose suitable electronic components based on the intrinsic electrical parameters of the TENG were summarized. This work provides valuable guidance for designing and fabricating self-powered IoT node devices.

## 1. Introduction

With the rapid development of IoT devices [1,2,3], the need to use renewable and environmentally friendly new energy sources to power them is also growing [4,5,6,7,8]. Utilizing the abundant, random, and irregular mechanical energy existing in the environment to power IoT devices would be a highly promising prospect [9]. Triboelectric nanogenerators, first proposed by ZhongLin Wang in 2012, offer a powerful technology to address this need [10,11]. TENGs are based on the principles of triboelectrification and electrostatic induction to harvest micromechanical vibration energy that is difficult to reuse [12]. At the same time, TENGs have attracted considerable attention from the research community due to their simple structure [13,14], high energy conversion efficiency [15], wide range of applications [16], and ease of fabrication [17]. To promote the development and practical application of this promising new technology, researchers have proposed various techniques to improve the output performance of TENGs. Some works have focused on improving the electrical output performance by combining TENG with mechanical structure design through cascading multiple TENGs and increasing the effective contact area of the TENG’s dielectric layer [18,19,20,21,22,23,24,25,26,27]. Other works have aimed to improve the electrical output performance of TENGs by modifying the dielectric layer material through physical and chemical methods to obtain a higher surface charge density of the dielectric layer [28,29,30,31,32]. However, when using TENG as a power supply device today, it is common to store energy before supplying power to the back-end consumer device. Therefore, the storable power density of TENGs is extremely important. In this work, a Z-shaped origami spring structure [33] and a buffer layer structure were adopted in a high storable power density TENG (HS-TENG) to make the frictional contact between the dielectric layers more complete and tighter, thus improving the output performance of the generator. More importantly, the basic electrical performance parameters of the HS-TENG ports were optimized using engineering methods [10,17,32] to match the circuit with the HS-TENG and to obtain a higher storable power density. The experimental data demonstrate that the HS-TENG exhibits an open-circuit voltage output of 645.17 V, a peak-to-peak value of the short-circuit current of 14.11 μA, and an external instantaneous power of 0.8 mW at an external impedance of 100 MΩ. These results indicate the favorable external power supply capability of the HS-TENG. Furthermore, the storable power density was measured to be 5.45 mW m^−2^ when an external 1 mF capacitor was connected. The proposed approach provides a novel TENG fabrication method and guidance for circuit component selection, which will serve as a reference for self-powered IoT node devices.

## 2. Materials and Methods

### 2.1. Fabrication of Experimental Testing Structure

**Figure 1:**
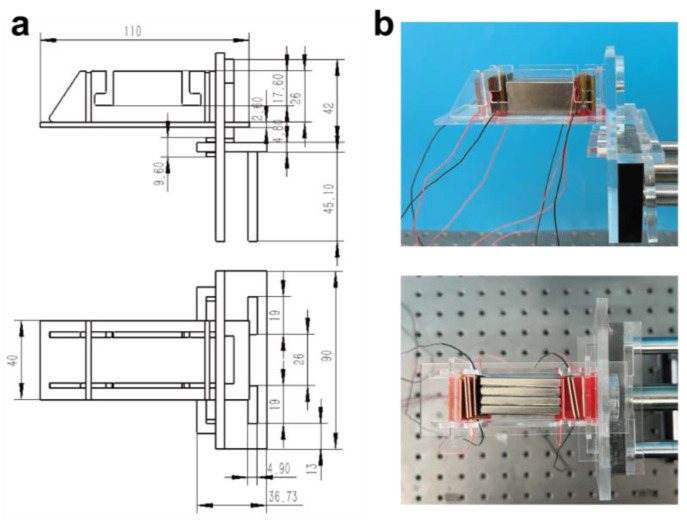
Experimental testing structure: (**a**) geometrical dimensions of the test structure (mm) and (**b**) optical photos of the test structure.

To eliminate the magnetic interference and gravitational acceleration on the experimental independent variable, the testing structure raises the TENG being tested away from the magnetic axis of the linear motor. Additionally, the overall testing structure is kept horizontal on the testing platform, with the TENG’s opening direction perpendicular to the direction of gravity. Three acrylic plates with a thickness of 5mm and sizes of 95 mm × 90 mm, 90 mm × 50 mm, and 36.73 mm × 90 mm were cut and joined to form a linear motor head clamp. Then, five acrylic plates with a thickness of 3 mm were used to make the TENG testing volume, which has three different sizes: 40 mm × 110 mm, 86 mm × 28.6 mm, and 40 mm × 27.6 mm. The HS-TENG is fixed on a size 40 mm × 27.6 mm bracket using 3M tape. The TENG testing volume is fixed with a pin structure and the linear motor head clamp, making it easy to replace during the testing process to ensure consistent testing conditions. The precise dimensions and assembly of the test structure are comprehensively depicted in Figure 1a, providing a detailed representation of its configuration. Figure 1b presents an optical photograph of the actual test structure, corresponding to the engineering drawing illustrated in Figure 1a.

### 2.2. Fabrication of the High-Storable Power Density TENG (HS-TENG)

The substrate of the HS-TENG, which includes FR-4, Brass, and Kapton, is made using a flexible circuit board process. A rectangular flexible circuit board with dimensions of 20 mm × 44 mm (one of the V-shaped parts of the HS-TENG) and a thickness of 0.3 mm was customized. The brass area is 20 mm × 20 mm, and it is connected to the back electrode via a small through-hole. A piece of 20 mm × 20 mm, 0.5 mm thick 3M-VHB tape is attached to the exposed brass electrode and used as a cushion layer. Then, a 30 μm piece of dual-conductive copper tape is used to cover the cushion layer, with one side covered by a 20 mm × 20 mm, 80 μm thick piece of FEP tape as the dielectric layer.

### 2.3. Fabrication of Power Management Circuit 

The rectifier bridge is a GOODWORK DB107 with a withstand voltage of 1 kV and a maximum rectification current of 1 A. When the current is 1 A, the diode voltage drop is 1.1 V. The high-voltage energy storage capacitor is a 1 nF 3 kV capacitor. The thyristor is an EC103M1 from Littelfuse with a maximum reverse voltage of 600 V. D1 diode is a SUNMATE 2EZ100D5 regulator diode, and D2 diode is a SUNMATE 2EZ200D5 regulator diode. The inductor is a PROD 100 μH 2 A straight insert type iron core inductor. The energy storage capacitor is a CX (ChengXing, Dongguan, China) 1000 μF 10 V aluminum electrolytic capacitor. The LTC3588 energy management module is connected in parallel with the electrolytic capacitor.

**Figure 2:**
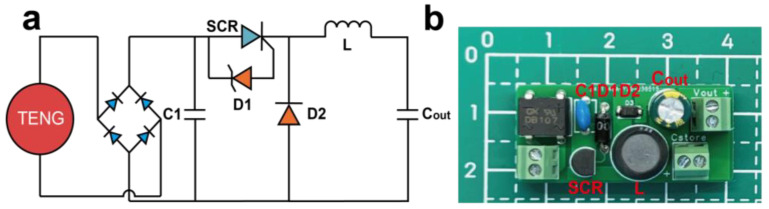
Power management circuit: (**a**) circuit topology and (**b**) optical photos of the power management circuit.

Figure 2 presents the circuit schematic and an actual optical photograph of the energy management circuit. Figure 2a depicts the fundamental circuit topology comprising ideal components, aiming to provide a clearer illustration of the energy transfer process and steps within the circuit. Figure 2b showcases the realized circuit based on the configuration shown in Figure 2a. The TENG energy input interface is positioned on the left, while the Cstore interface is to facilitate the increase of storage capacitance. The Cout capacitor and Cstore interface are parallel circuit configuration. Finally, the energy output port is denoted as Vout.

The energy harvesting circuit must minimize its energy consumption to enhance energy conversion efficiency. Regarding the rectifier bridge, the selection of a low voltage-drop rectifier bridge, based on the TENG’s short-circuit current, is essential. This approach represents a straightforward and effective means of reducing energy conversion losses. Due to the presence of C1’s leakage current and parasitic resistance, the storage time of energy on the C1 capacitor needs to be sufficiently short. Additionally, the release voltage of the C1 capacitor is determined by the voltage regulation value of the D1 Zener diode, whereby a lower release voltage corresponds to a shorter storage time in C1. Nevertheless, it is important to note that an excessively low voltage regulation value for D1 can lead to continuous conduction of the SCR, thereby impeding the circuit’s ability to achieve voltage reduction and current amplification.

Engineering calculation method is based on the TENG parameters Q_sc_, I_sc,_ and V_pp_. (Q_sc_: TENG short circuit transfer charge; I_sc_: TENG short circuit current; and V_pp_: TENG open circuit peak-to-peak voltage.)

(1) $$Q_{\mathrm{sc}}=\frac{C_{1}U_{ZT}}{n}$$

(2) $$U_{ZT}=\frac{1}{2}U=\frac{1}{2}\times 0.9\times \frac{V_{pp}}{2}$$

(3) $$t_{on}=\frac{n}{2f}$$

U: margin half-peak voltage; U_ZT_: D1 diode regulation value; t_on_: SCR conduction time; and n: number of work cycles. The number of work cycles represents the frequency of the TENG’s output alternating voltage signal, as well as the desired energy discharge magnitude. Determining this value necessitates a comprehensive consideration of factors such as the TENG’s operating frequency, the energy storage time of capacitor C1, the saturation current value of inductor L, and the equivalent DC resistance. In the case of the HS-TENG, which operates in the contact-separation mode, the frequency of the alternating voltage signal is generally the lowest compared to TENGs in other modes. To prevent excessive energy waste in the C1 capacitor caused by an excessively long “t_on_” duration, a value of n is set to 10. Furthermore, the use of 0.9 times the half peak value in the formula represents an engineering approximation, while multiplying it by 1/2 provides a sufficient margin to avoid the breakdown of the Zener diode and enable the conduction signal for the SCR.

### 2.4. Electrical Measurement and Characterization 

The testing structure is driven by a linear motor (LinMot BF01-37). The transferred charge and current are measured by an electrostatic meter (KEITHLEY 6517B). It should be noted that since the TENG voltage exceeded the measurement range of the electrostatic meter, an attenuator probe (PINTECH HVP-40) is used to measure the voltage after attenuation, with an attenuation ratio of 1000 times. To ensure the accuracy of the open-circuit voltage, an oscilloscope (RIGOL MSO8204) and a 100:1 oscilloscope probe (P4100 100 MΩ 6 pF) was used to measure the open-circuit voltage of the HS-TENG, which was consistent with the measurements obtained by an electrostatic voltmeter and an attenuated probe.

## 3. Results and Discussion 

**Figure 3:**
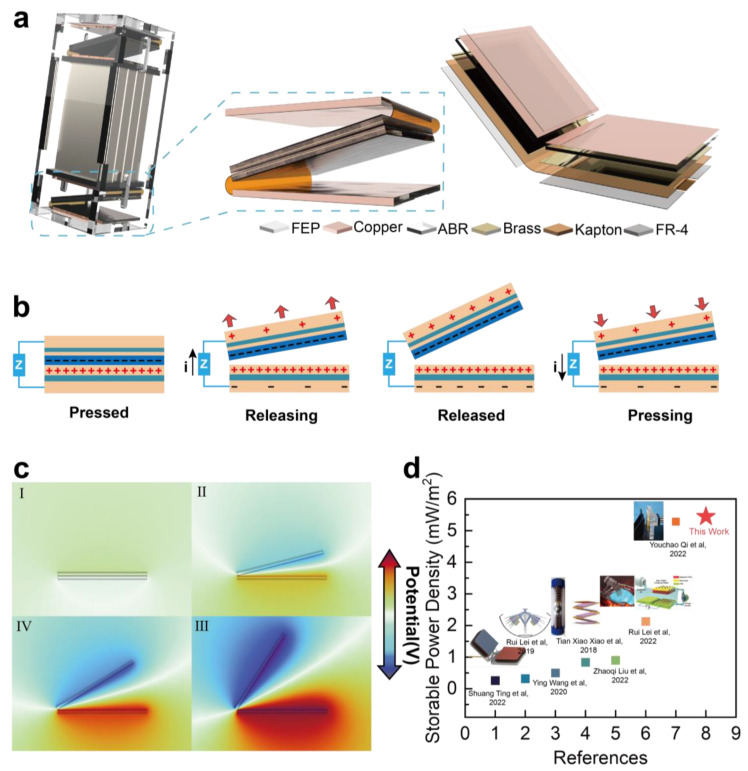
Illustrates the design of the centimeter-scale HS-TENG and its comparison in storable power density. (**a**) shows a rendered view of the HS-TENG test structure, including the multilayered materials used in its construction. (**b**) presents a simplified working principle of the HS-TENG basic unit. (**c**) displays the COMSOL simulation of the open-circuit voltage potential distribution for a single V-shaped structure of the HS-TENG. figure I Pressed state, figure II and III Releasing state at different angle, figure IV Released state. (**d**) presents a comparison of storable power density for different works [34,35,36,37,38,39,40].

This study proposes a high storable power density triboelectric nanogenerator. Figure 3 illustrates the fundamental constituent materials of the multilayer TENG and presents the 3D model of the experimental setup. The figure provides a succinct overview of the generator’s basic operating principle and showcases its potential distribution on the simulation surface. Moreover, it highlights the scientific advancements accomplished in this study.

The HS-TENG full 3D view is shown in Figure 3a. The diagram includes the test structure, the basic unit of the folded spring, and the multilayered structure of the HS-TENG. The test structure is assembled using acrylic plates, with two identical HS-TENG basic units placed in the upper and lower spatial locations to minimize experimental errors resulting from randomization. The basic unit of the folded spring is formed by bonding two small V-shaped structures, which allows for better utilization of both sides of the same physical location, thereby improving the spatial utilization efficiency of the HS-TENG. The small volume and modular design of the basic units offer considerable flexibility for assembling various configurations and cascades of the TENG. By combining different quantities of TENG basic units, a larger cascade unit can be constructed to yield enhanced output performance in diverse application scenarios. This fundamental interconnection approach enables remarkable scalability for the HS-TENG. 

The multilayered metal structure in the middle of the test structure functions as a weighting block to investigate the effect of different numbers of mass blocks on the output during the compression of HS-TENG. In the far-right part of Figure 3a, a detailed display of the multilayered structure of the HS-TENG is provided, consisting of fluorinated ethylene propylene (FEP), copper, Propylamine (ABR), brass, Kapton, and FR-4 as the constituent materials of the HS-TENG. FEP and copper act as the dielectric layers, ABR serves as a cushion layer to ensure full contact between the dielectric layers, brass provides a large number of free electrons for the induction electrode, and FR-4 serves as a supportive structure for these relatively soft materials. By using the aforementioned structure and stacking multiple layers of materials, the goal of achieving good output performance in small volumes and space is achieved. 

Figure 3b presents a simplified working principle of the HS-TENG’s V shape unit, which can be abstracted into four stages: pressed, releasing, released, and pressing. When there is a load between the two induction electrodes, during the releasing stage, the potential difference between the two electrodes drives electrons from the upper electrode to the lower electrode, generating an upward instantaneous current at the moment of release. As the distance between the two dielectric layers decreases during pressing, the potential of the upper electrode becomes higher than the lower electrode, causing electrons to flow back from the lower electrode to the upper electrode, reducing the amount of induced charge on the electrodes and generating a downward instantaneous current. When the dielectric layers come into contact again, all the induced charges are neutralized. Figure 3c shows the spatial distribution of voltage potential corresponding to the above four operating modes through COMSOL simulation without a load. A multilayered model of the actual structure is added to the COMSOL simulation to better fit the real composition of HS-TENG. The specific simulation parameters and the detailed division of the simulation region are presented in Figure S1. The animation of the simulation process is included in Supporting Information Video S1.

Figure 3d presents, in a chart format, the works of seven previous studies in increasing order of storable power density. The first four studies mainly employed the combination of mechanical structures with TENG to improve the generator’s output performance. The fifth study modified the dielectric layer material by physical means to obtain a higher surface charge density and thus improve the generator’s output performance. The sixth study added oil as a medium between two dielectric layers to enhance the output performance. The seventh study used a method of ferromagnetic-based charge accumulation and, with the use of energy management circuits, improved the TENG’s output performance. To enable a comprehensive comparison with the output performance of the aforementioned seven references, a comparison dataset is introduced for the current application scenario, focusing on storable power density. Storable power density signifies the quantity of energy stored in the capacitor, available for powering the intended load. It is determined by calculating the actual energy stored in the capacitor, the time required to accumulate this energy, and the effective dielectric layer area of the TENG. Formula 1 in the supporting information provides the means to calculate the storable power density. Specific values and parameters of the mentioned references can be found in Supporting Information Table S1. While previous studies have made significant strides in enhancing the electrical output performance of TENG for portable applications, they have not addressed the optimization of storable energy utilization in practical scenarios. This work combines previous research efforts and proposes a solution to address the issue of storable energy supply in practical applications, such as self-powered Internet of Things (IoT) nodes. Compared to the reference works, this study achieved a certain improvement in the parameter of storable power density.

**Figure 4:**
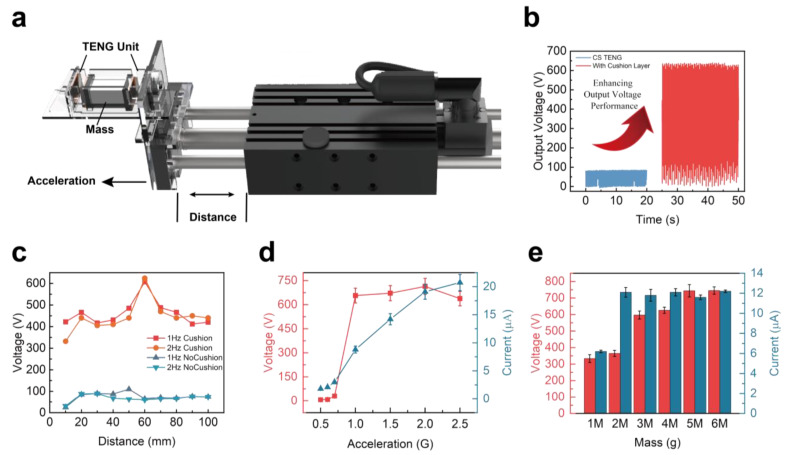
The electrical characteristics and parameter optimization of a single TENG. (**a**) A linear motor test platform with annotated testing parameters. (**b**) A comparison between a single Z-shaped TENG with and without a cushion layer. (**c**) Open-circuit voltage of TENG with and without a cushion layer at different movement distances. (**d**) Voltage-current characteristics of TENG under various accelerations. (**e**) Voltage-current characteristics of TENG with different vibration mass blocks.

Figure 4 illustrates the fundamental test environment of the HS-TENG and the variable parameters employed in the experiment. The figure presents the electrical performance parameters, both with and without considering the output voltage difference originating from the cushion layer. Additionally, it investigates the influence of distance, acceleration, and mass parameters on electrical performance.

To avoid the interference of the linear motor’s magnetic axis on the test device during the experimental process, a structure shown in Figure 4a was employed to investigate the parameters affecting the output of the HS-TENG. During the testing phase, the two TENGs were positioned in a parallel orientation to the test bench to prevent any potential inconsistencies in their electrical performance due to gravitational influence. The acceleration depicted in the figure represents the acceleration of the linear motor applied to the test structure, rather than the acceleration acting on the HS-TENG itself. Similarly, the distance measurement refers to the displacement of the motor head relative to the fixed section of the testing platform, and not to the movement distance of the HS-TENG’s dielectric layer. 

Figure 4b presents the open-circuit voltage characteristics of a spring structure TENG with and without a cushion layer. The average peak-to-peak output voltage of the origami TENG without a cushion layer was 85.37 V, while adding a cushion layer increased the average peak-to-peak output voltage to 638.25 V, resulting in a clear 7.5-fold increase in open-circuit voltage. The cushion layer softened the dielectric layer, making it more elastic and thicker, resulting in an increase in the effective dielectric contact area compared to the TENG without a cushion layer for the same displacement. 

Figure 4c shows the average peak-to-peak open-circuit voltage values at intervals of 10 mm with the change in the moving distance of the testing structure at frequencies of 1 Hz and 2 Hz. It is noteworthy that the distance in the figure does not represent the change in the dielectric layer of the TENG, but rather the external vibration distance of the testing structure as an excitation and the output voltage of the TENG as a response, which is shown as a line graph. The maximum open-circuit voltage output point of the TENG without a cushion layer was 109.25 V at a moving distance of 50 mm under 1 Hz and 89.94 V at a moving distance of 30 mm under 2 Hz. However, the overall change was not significant under the influence of the displacement. For the TENG with a cushion layer, the maximum open-circuit voltage at frequencies of 1 Hz and 2 Hz occurred at a moving distance of 60 mm, reaching 618.55 V. The TENG with a cushion layer was more sensitive to external excitation movement. Nevertheless, the overall test results showed that the introduction of a cushion layer improved the open-circuit voltage output performance of the TENG.

Figure 4d displays the curves of open-circuit voltage and short-circuit current under different accelerations, where G represents the gravitational acceleration of 9.8 m/s^2^. When the acceleration of the linear motor was greater than 0.6 G, it could drive the vibration mass block to work the TENG normally. As the acceleration increased, the voltage reached 658.38 V at 1 G acceleration, with a fluctuation not exceeding ±10%. After reaching 1 G acceleration, the peak-to-peak voltage fluctuated only slightly with the acceleration change. The current value increased gradually with the increase in acceleration, but the rising trend gradually became slower. Hence, it is evident that the HS-TENG is capable of performing efficiently under external accelerations within the range of 0.7–2 G. However, it is noteworthy that a higher proportion of mechanical vibration energy is converted into electrical energy within the acceleration range of 0.7–1.6 G. 

The impact of the cushion layer on the output of TENG is significant. To investigate the effect of cushion layer thickness on the electrical output performance of a given TENG size, five sets of experiments were conducted, and the corresponding data are presented in Figure S2. As depicted in Figure S2a, the cushion layer with a thickness of 0.5 mm exhibits better output performance under an acceleration of 1.5–2 G. Furthermore, the voltage is less affected by acceleration when compared to the current, but an increase in acceleration beyond 2 G results in a slowdown of the current’s raising rate of change. Figure S2b illustrates the electrical output performance of different accelerations and thicknesses. The overall trend of the voltage is less affected by frequency, and the TENG with the same thickness as the cushion layer generally maintains a stable voltage output. In contrast, the current increases significantly with the frequency, which is consistent with the output characteristics of a typical TENG. Figure S2c shows the variation in TENG output performance with different vibrating masses and cushion layer thicknesses. Notably, the current output is significantly higher for a cushion layer thickness of 0.5 mm than for the other two thicknesses. Moreover, the added cushion layer’s capacitance at different excitation frequencies is presented in Figure S2d, while Figure S2e summarizes the maximum output characteristics of three cushion thicknesses. Overall, the cushion layer with a thickness of 0.5 mm offers a significant advantage in electrical performance.

Figure 4e investigates the electrical output performance of the HS-TENG by changing the number of mass blocks under the condition of a moving distance of 60mm and an acceleration of 1.6 G (1 M mass block is equivalent to 25.2 g). The primary change in the electrical output performance of the HS-TENG occurred in the interval of 1 M to 2 M mass blocks. The subsequent increase in mass blocks had little effect on the current output performance of the generator. However, the voltage output performance gradually increased with an increase in the number of mass blocks until the voltage change could be almost negligible after adding 5 M mass blocks.

**Figure 5:**
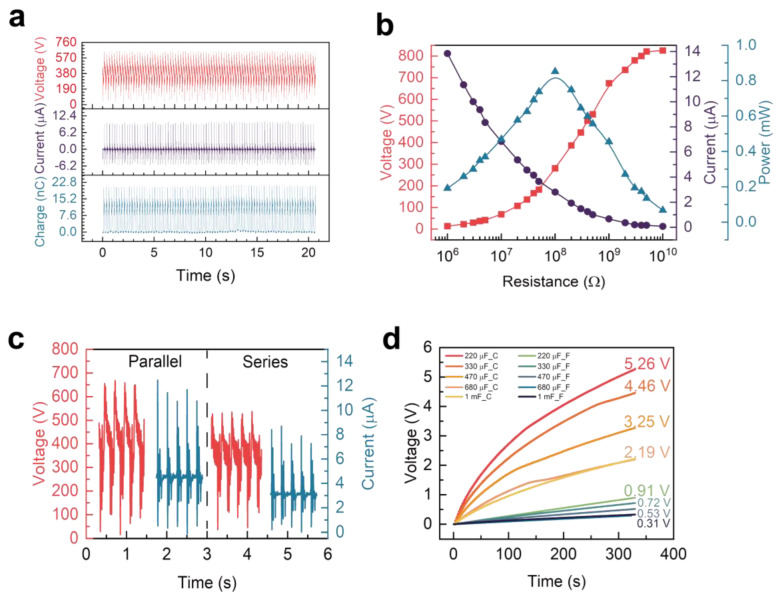
The output performance of the HS–TENG. (**a**) The voltage, current, and charge quantity of HS–TENG. (**b**) The real part of the impedance matching curve of the HS–TENG. (**c**) The voltage and current waveforms of the dual HS–TENG in series–parallel circuit. (**d**) The charging capacitor of the rectifier bridge and power management circuit of the HS–TENG.

When testing the electrical performance of the HS-TENG with a displacement of 60 mm, acceleration of 1.6 G, and a vibrating mass block of 4 M, Figure 5a presents the voltage, current, and charge of the HS-TENG. The HS-TENG exhibited a peak-to-peak voltage of 645.17 V, a peak-to-peak current of 14.11 μA, and a transfer charge of 20.05 nC in a working cycle. Impedance matching is a crucial parameter for TENG to supply power to the load output. This process involves taking the conjugate of the circuit port characteristics of TENG, where the real part is equal and the imaginary part is opposite to each other. In practical engineering, it is often approximated that the real part is equal, and the influence of the imaginary part is neglected, which can still achieve a better power output effect [41]. Figure 5b shows the test range of load resistance values from 1 MΩ to 10 GΩ to clearly observe the maximum power matching output point of 0.852 mW at 100 MΩ.

In Figure 5c, the investigation is centered on the optimal connection method for the two basic Z-shaped units of HS−TENG to achieve higher output. The graph clearly depicts that the parallel connection of the units provides better output performance than the series connection. This is because the phase and frequency of the two TENGs may not be entirely matched during the working process. This mismatch could cause the output voltage of one TENG to be at the positive half-peak while the output voltage of the other TENG is at the negative half-peak, leading to a cancellation of electric charges and a weakened output voltage. When TENGs are connected in parallel, the circuit port characteristic of TENG, which approximates the capacitance to an open circuit for low-frequency signals, ensures that the transferred charge will not be consumed by another generator, even if the phases do not match.

Figure 5d compares the energy collection effects of a full-bridge rectifier and a capacitor-collecting generator with energy management circuits. It is evident from the graph that the energy collection effect of the energy management circuit is significantly better than that of the full-bridge rectifier, as the 1 mF capacitor can be quickly charged to 2.19 V in only 330 s, compared to 0.31 V with the full-bridge rectifier. The charging speed of the energy management circuit is improved by seven times.

**Figure 6:**
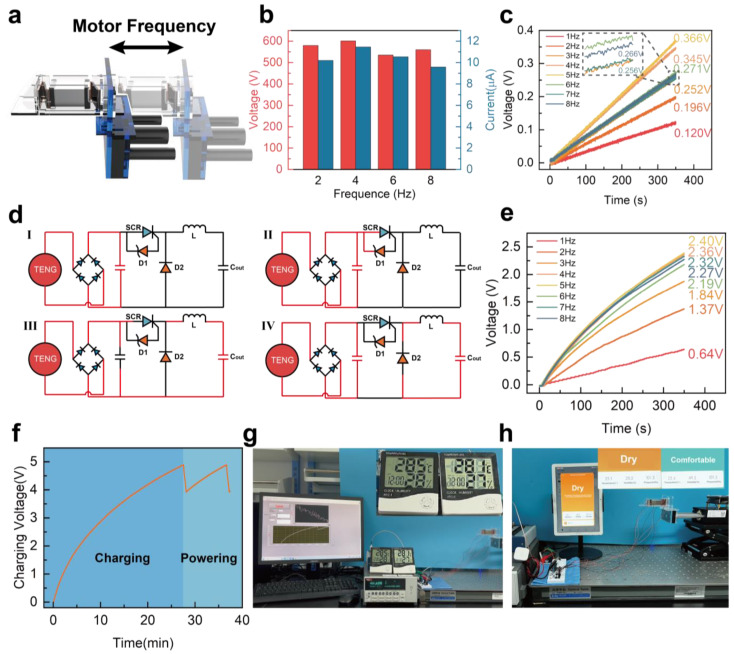
Management circuit of HS−TENG. (**a**) Schematic diagram of HS−TENG frequency test. (**b**) Basic electrical performance of HS−TENG. (**c**) Charging capacity curve of dual HS−TENGs using FBR at different operating frequencies. (**d**) Schematic diagram of the four-stage operation of the management circuit. (**e**) Charging capacity curve of dual HS−TENGs after using the management circuit. (**f**) Working curve of 1 mF capacitor when driving the load. (**g**) HS−TENG supplying power to two temperature and humidity sensors. (**h**) HS−TENG driving the operation of the wireless sensing node.

Figure 6 presents an investigation into the performance of HS−TENG output and the comparison of the capacitor charging speed of HS−TENG at different operating frequencies. The testing structure and frequency testing schematic are shown in Figure 6a, with testing conditions controlled at an acceleration of 1.6 G and a displacement of 30.625 mm. Figure 6b reveals that the open-circuit voltage and short-circuit current of HS−TENG are relatively insensitive to frequency, indicating a good output response of HS−TENG to low-frequency mechanical signals in the testing structure. Figure 6c shows that the energy stored in the capacitor generated by HS−TENG through the full-bridge rectifier is small, as most of the energy is consumed during transmission. To improve the efficiency of HS−TENG during the energy transmission stage, the basic topology structure of the BUCK step-down technology circuit is used in this work.

On the basis of the original circuit topology, the electrical output signal of the HS−TENG port is analyzed. The method described in Materials and Methods is used to optimize the suitability of this circuit for HS−TENG and achieve higher energy conversion efficiency. Figure 6d illustrates the four stages of circuit operation: in the first stage, HS−TENG charges the high-voltage capacitor through the full-bridge rectifier, completing the first stage of energy storage; in the second stage, when the voltage of the first-stage energy storage capacitor reaches the breakdown voltage threshold of the D1 voltage regulator diode, the voltage regulator diode breaks down, and the silicon-controlled rectifier (SCR) receives a passage signal, quickly releasing the energy in the first-stage energy storage capacitor to the downstream circuit; in the third stage, the controlled SCR is turned off, and the first-stage energy storage capacitor re-enters the energy storage stage, during which the diode can prevent large currents and over voltages from causing a breakdown of the downstream aluminum electrolytic capacitor, and the inductor can convert the energy into a magnetic field energy storage; in the fourth stage, the D2 diode conducts in the forward direction, transferring the stored energy from the inductor L to the storage capacitor Cout. At this stage, detailed component selection for the circuit is conducted, taking into account the performance of the TENG output and the requirement to maintain the high energy conversion efficiency of the BUCK circuit. 

Figure 6e shows the relationship between the charging curve of the 1 mF capacitor with the energy management circuit and the frequency. The circuit can reach the maximum energy storage output point when the frequency of the testing structure reaches 5 Hz. The circuit structure described herein is integrated with the LTC3588 ultra-low-power energy harvesting power management module to provide a stable 3.3 V power supply voltage to the circuit load and manage the energy harvesting strategy. Specifically, the module enables the provision of power to the load circuit once the voltage stored in the storage capacitor reaches 5 V. Conversely, the module discontinues the power supply to the load circuit once the voltage stored in the storage capacitor drops to 4 V, thus allowing the energy storage capacitor to recharge.

Figure 6f shows the performance curve of the energy storage capacitor under load. This demonstrates the HS-TENG’s ability to drive sensors at a minute level in vibrating environments. Often, other similar-size TENG drive sensors take longer to operate once. Figure 6g demonstrates the power supply capability of the circuit, which can support the normal operation of two temperature and humidity sensors for several seconds. A video of the testing process is in Supporting Information Video S2. Moreover, in Figure 6h, this system can drive the Xiaomi Mi (Beijing, China) Home wireless temperature, humidity, and pressure sensors to achieve wireless sensing every ten minutes or so. A video of the testing process is in Supporting Information Video S3. Overall, Figure 6 presents the testing and results of HS−TENG output performance and the charging rate of the power management circuit. The proposed circuit structure shows efficient energy management and stable power supply voltage to the load circuit. The results also demonstrate the capability of the system to drive various sensors for wireless sensing applications.

## 4. Conclusions

In summary, this study investigates sensor self-power by employing an origami spring structure and Buck energy harvesting circuit topology. A multilayer stacked TENG structure is utilized, and the selection of circuit component parameters is optimized. The optimal output performance of the harvesting device is determined by considering the multilayer structure, external disturbance distance, external disturbance acceleration, device vibrator mass, and device operating frequency. Through these specific processes, the storable power density of the HS-TENG is significantly enhanced, offering valuable insights for micro-vibration energy harvesting units and TENG electric energy harvesting circuits. The key conclusions drawn from this study are as follows:

(1) The manufacturing process of the TENG incorporates the use of Flexible Printed Circuit Board (FPCB) technology and includes an interlayer cushion layer. This design enhancement results in improved electrical performance of the HS-TENG compared to TENGs of the same volume size.
(2) Engineering optimization approximation methods are employed, analyzing the workflow of the circuit and proposing a component selection approach for the energy harvesting circuit specifically applicable to the HS-TENG based on experimental results. This approach leads to an increased storable power density, aligning with the electrical consumption requirements of sensors and Internet of Things (IoT) nodes.

## Data Availability

The data presented in this study are available on request from the corresponding author.

## Supplementary Materials

The following supporting information can be downloaded at https://www.mdpi.com/article/10.3390/ma16134669/s1. Figure S1: COMSOL simulation details; Figure S2: The electrical output performance corresponding to different buffer layer thicknesses, and the measurement of buffer layer capacitance; Formula 1: Calculation formula for storable power density; Table S1: Comparative table of storable power density parameters; Video S1: COMSOL simulation of voltage potential; Video S2: HS-TENG supplying two temperature sensors; Video S3: HS-TENG driving wireless sensing node.
