# Supplementary material for: High Storable Power Density of Triboelectric Nanogenerator within Centimeter Size
Source: Materials (Basel). 2023 Jun 28;16(13):4669. doi: 10.3390/ma16134669 (PMC10342295; doi:10.3390/ma16134669)
Supplement: Supplementary file 1 [file materials-16-04669-s001.zip › materials-2430044-supplementary.pdf]

Figure S1 comsol simulation details

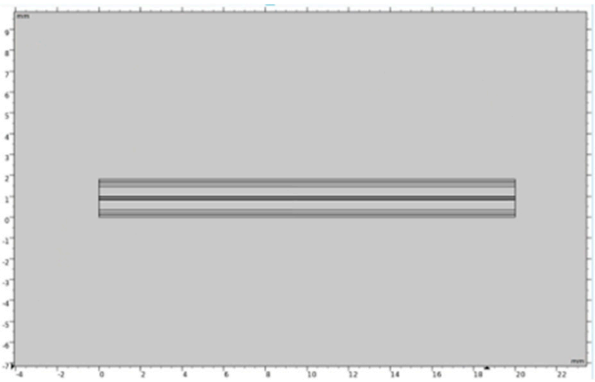

Materials	Relative permittivity
Air	1
FEP	2.1
Brass	1
Copper	1
ABR	3.5
FR-4	4.5

Figure S2 The electrical output performance corresponding to different buffer layer thicknesses, as well as the measurement of buffer layer capacitance

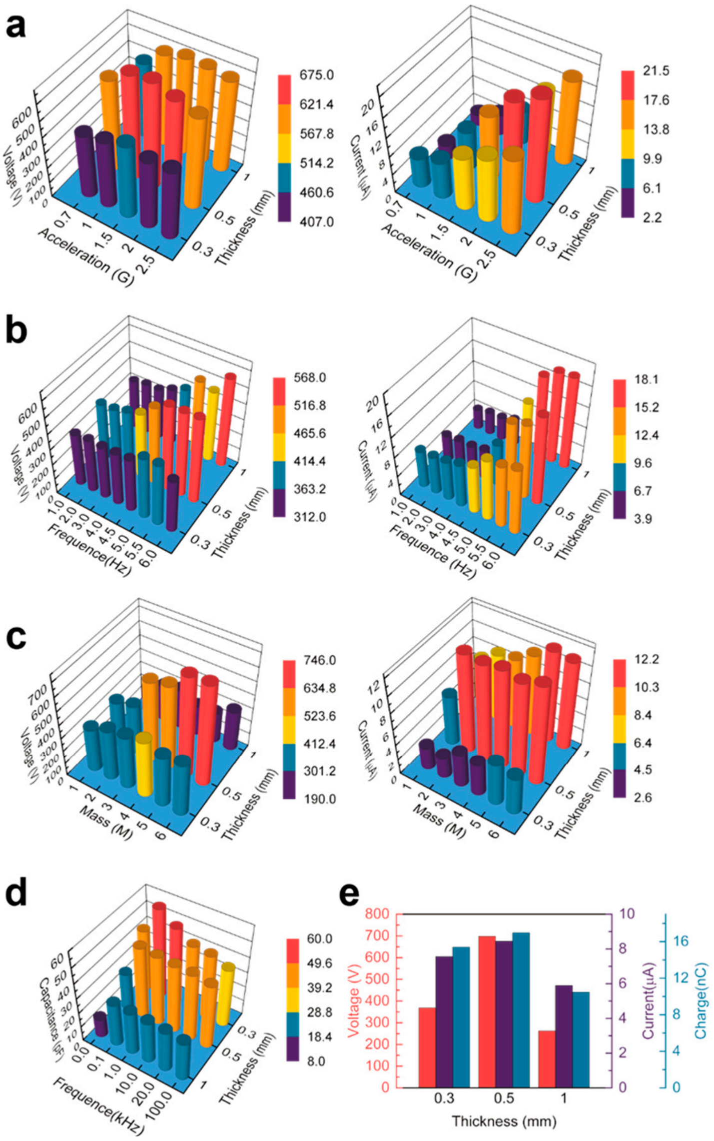

Calculation formula for storable power density:

$$E = \frac{1}{2} cu^2 (J); P = \frac{E}{t} (w); PD = \frac{P}{S} (w/m^2)$$

E represents stored energy; C is the storage capacitance value; U is the voltage value on the storage capacitor; P is the power; T is the working hours; S represents the effective friction area of the actual dielectric layer; PD represents power density.

Table S1: Comparative table of storable power density parameters

References	C(F)	U(V)	t(s)	S(m^2)	PD(mW/m^2)
This work	1m	2.40	330	1.6e-3	5.4545
1	3.3m	3	3600	0.016	0.2578
2	470u	2.3	300	0.013	0.31876
3	1u	15	25	9e-3	0.5
4	470u	1.6	160	4.5e-3	0.8356
5	1u	6	200	1e-4	0.9
6	1m	3.2	200	0.012	2.1333
7	330u	4.6	275	1.8e-3	5.29
